# Endogenously produced LG3/4/5-peptide protects testes against toxicant-induced injury

**DOI:** 10.1038/s41419-020-2608-8

**Published:** 2020-06-08

**Authors:** Linxi Li, Baiping Mao, Siwen Wu, Huitao Li, Lixiu Lv, Renshan Ge, C. Yan Cheng

**Affiliations:** 1 The Second Affiliated Hospital and Yuying Children’s Hospital, Wenzhou Medical University, 325027 Wenzhou, Zhejiang China; 2 The Mary M. Wohlford Laboratory for Male Contraceptive Research, Center for Biomedical Research, Population Council, 1230 York Ave, New York, NY 10065 USA

**Keywords:** Cell proliferation, Drug development

## Abstract

Laminin-α2 chain is one of the major constituent proteins of the basement membrane in the mammalian testis. The laminin-type globular (LG) domains of LG3, 4 and 5 (LG3/4/5, an 80 kDa fragment) can be cleaved from laminin-α2 chain at the C-terminus via the action of matrix metalloproteinase 9 (MMP-9). This LG3/4/5 is a biologically active fragment, capable of modulating the Sertoli cell blood–testis barrier (BTB) function by tightening the barrier both in vitro and in vivo. Overexpression of LG3/4/5 cloned into a mammalian expression vector pCI-neo in Sertoli cells in a Sertoli cell in vitro model with a functional BTB also protected Sertoli cells from cadmium chloride (CdCl_2_, an environmental toxicant) mediated cell injury. Importantly, overexpression of LG3/4/5 in the testis in vivo was found to block or rescue cadmium-induced BTB disruption and testis injury. LG3/4/5 was found to exert its BTB and spermatogenesis promoting effects through corrective spatiotemporal expression of actin- and MT-based regulatory proteins by maintaining the cytoskeletons in the testis, illustrating the therapeutic implication of this novel bioactive fragment.

## Introduction

Recent studies have shown that laminin-α2 chain (formerly called merosin), one of the major constituent proteins of the basement membrane in the testis^1–4^, can be cleaved by matrix metalloproteinase 9 (MMP-9) to generate a biologically active 80 kDa fragment^5^. This laminin-α2 80 kDa fragment containing the LG3/4/5 domain, generated at the basement membrane from laminin-α2 chain, was found to be transported to the apical ES, a testis-specific anchoring junction that confers spermatid adhesion to Sertoli cells in the seminiferous epithelium^6,7^, through a microtubule (MT)-dependent transport mechanism^5^. More important, this 80 kDa fragment was shown to promote Sertoli cell blood–testis barrier (BTB) integrity, making the barrier “tighter”^5^. It exerts its biological effects through the mTORC1/rpS6/Akt1/2 signaling pathway^8^. The findings based on these studies thus illustrate the presence of a local physiological axis between the basement membrane and the BTB, wherein the laminin-α2 80 kDa fragment acts as an autocrine peptide^5,8^. Furthermore, studies have shown that there is also a functional axis that links the BTB and apical ES designated the apical ES-BTB axis wherein a locally produced F5-peptide generated from the laminin-γ3 chain (an apical ES-specific adhesion protein^9–11^), which serves as another autocrine peptide that promotes BTB disruption, making the barrier “leaky”^12–15^. In short, this 80 kDa fragment is a novel regulatory peptide in the apical ES-BTB-basement membrane that is working in concert with the F5-peptide to modulate and coordinate cellular events across the seminiferous epithelium to support the epithelial cycle of spermatogenesis. The concept of this 80 kDa fragment derived from the laminin-α2 chain that also regulates apical ES function is consistent with earlier reports using a genetic model in which knockout (KO) of laminin-α2 chain in mice was found to induce extensive disruption of the apical ES^16^, leading to elongate spermatid exfoliation from the testis^17^. This observation thus implicates that a loss of basement membrane function through deletion of laminin-α2 chain impedes apical ES function at the other end of the seminiferous epithelium. In this context, it is of interest to note that deletion of laminin-α2 chain led to muscular dystrophy in mice, making this a novel model to study merosin (i.e., laminin-α2)-deficient congenital muscular dystrophy^16^ since mutations in the laminin-α2 gene leads to muscular dystrophy in humans and mice^18,19^. However, it remains to be examined if mutations of laminin-α2 chain that led to congenital muscular dystrophy in humans was associated with male infertility. In order to unravel the physiological significance of the LG3/4/5 fragment in the testis, we sought to clone this laminin-α2 fragment for its overexpression in the testis to examine if this biologically active fragment promotes spermatogenesis, besides making the BTB “tighter”, using a toxicant-induced testis injury model. Interestingly, this LG3/4/5 fragment was shown to protect the testis from cadmium-induced testis injury, and also capable of reversing cadmium-induced male reproductive dysfunction, illustrating its therapeutic potential in the treatment of male infertility.

## Materials and methods

### Animals

Adult Sprague-Dawley rats at ~250 gm b.w. and male pups at 16 or 17 days of age were purchased from Charles River Laboratories (Kingston, NY). Adult rats were housed in groups of two per cage. Each ten male pups were housed with a foster mother in the same cage. All animals were kept at the Rockefeller University Comparative Bioscience Center (CBC) with free access to water and standard rat chow and water ad libitum in a light–dark cycle of 12 h each at 21 ± 1^o^C. The use of animals and recombinant DNA materials including different cDNA constructs (i.e., plasmid DNA) and detailed experimental protocols for in vitro and in vivo experiments was approved by the Rockefeller University Institutional Animal Care and Use Committee (IACUC) with Protocol Numbers 15-780-H and 18-043-H and Rockefeller University Institutional Biosafety Committee (IBC) with Protocol Number 2-15-04-007, respectively. Rats were euthanized by CO_2_ asphyxiation using slow (20–30%/min) displacement of chamber air from compressed carbon dioxide in a euthanasia chamber built and approved by the Rockefeller University Laboratory Safety and Environmental Health (LESH).

### Primer sequences and antibodies

Nucleotide sequence information on primers used to obtain different cDNA constructs are listed in Table 1. Antibodies used for different experiments in this report were obtained commercially unless otherwise specified (Table 2). The Research Resource Identifier (RRID) information and working dilutions of antibodies used for our experiments are listed in Table 2.

**Table 1:** Primers used for RT-PCR and gene cloning to obtain different cDNA constructs for various experiments in this report.

	Primer sequences			
Gene fragment		Nucleotide position	Length (bp)	Annealing Temp (^o^C)
(1) LG3 (sense)	5′-CCG***CTCGAG***ATGGTCGGAACGGAAATCAACCTG-3′	7741-7761	468	66
(2) LG3 (antisense)	5′-ACGC***GTCGAC****TTA*AGCTATAGGCTGTGCAAAGTC-3′	8188-8208		
(3) LG4 (sense)	5′-CCG***CTCGAG***ATGAAAAACCGCCTCACCATTGAG-3′	8455-8475	429	70
(4) LG4 (antisense)	5′-ACGC***GTCGAC****TTA*GGTAGGCTGGTCCAGATCAAC-3′	8863-8883		
(5) LG5 (sense)	5′-CCG***CTCGAG***ATGGTTGGATTGGACCTTCTTGTA-3′	8977-8997	456	66
(6) LG5 (antisense)	5′-ACGC***GTCGAC****TTA*CAGAGCCTTGGCAAAATTAAC-3′	9412-9432		
(7) LG3/4 (sense)	5′-CCG***CTCGAG***ATGGTCGGAACGGAAATCAACCTG-3′	7741-7761	1143	70
(8) LG3/4 (antisense)	5′-ACGC***GTCGAC****TTA*GGTAGGCTGGTCCAGATCAAC-3′	8863-8883		
(9) LG4/5 (sense)	5′-CCG***CTCGAG***ATGAAAAACCGCCTCACCATTGAG-3′	8455-8475	978	68
(10) LG4/5 (antisense)	5′-ACGC***GTCGAC****TTA*CAGAGCCTTGGCAAAATTAAC-3′	9412-9432		
(11) LG3/4/5 (sense)	5′-CCG***CTCGAG***ATGGTCGGAACGGAAATCAACCTG-3′	7741-7761	1692	70
(12) LG3/4/5 (antisense)	5′-ACGC***GTCGAC****TTA*CAGAGCCTTGGCAAAATTAAC-3′	9412-9432		

The primer sequences used for obtaining different cDNA constructs were obtained from Genbank with Accession Number: XM_017590489.1 for rat laminin-α2 chain. Restriction sites are *Xho*I (***CTCGAG***) in the sense primer corresponding to the 5′-end, and *Sal*I (***GTCGAC***) in the antisense primer corresponding to the 3′-end. The start codon of ATG (sense) is underlined. The stop codon of *TTA* (antisense) is italicized and underlined.

**Table 2:** Antibodies used for different experiments in this report.

Antibody (RRID No.)	Host Species	Vendor	Catalog number	Working dilution IF
Dvl3 (AB_1841289)	Rabbit	Sigma-Aldrich	WH0001857M4	1:250
CAR (AB_10915738)	Mouse	Santa Cruz Biotechnology	sc-373791	1:50
ZO-1 (AB_2533938)	Rabbit	Thermo Fisher Scientific	61–7300	1:100
N-cadherin (AB_2313779)	Mouse	Thermo Fisher Scientific	33–3900	1:100
β-catenin (AB_2533982)	Rabbit	Thermo Fisher Scientific	71–2700	1:100
Eps8 (AB_397544)	Mouse	Thermo Fisher Scientific	610143	1:100
Arp3 (AB_476749)	Mouse	Sigma-Aldrich	A5979	1:100
MARK4 (AB_2636847)	Rabbit	Proteintech Group	20174-1-AP	1:50
EB1 (AB_2141629)	Rabbit	Santa Cruz Biotechnology	sc-15347	1:300
α-tubulin (AB_2241126)	Mouse	Abcam	ab7291	1:200
Detyrosinated α-tubulin (AB_869990)	Rabbit	Abcam	ab48389	1:200
Rabbit IgG-Alexa Fluor 488 (AB_2576217)	Goat	Thermo Fisher Scientific	A-11034	1:250
Mouse IgG-Alexa Fluor 488 (AB_2534088)	Goat	Thermo Fisher Scientific	A-11029	1:250
Rabbit IgG-Alexa Fluor 555 (AB_141784)	Goat	Thermo Fisher Scientific	A-21428	1:250
Mouse IgG-Alexa Fluor 555 (AB_141780)	Goat	Thermo Fisher Scientific	A-21424	1:250

*Arp3* actin-related protein 3, which together with Arp2 create the Arp2/3 complex known to induce branched actin polymerization, converting linear actin filaments into a branched network; *CAR* coxsackievirus and adenovirus receptor, a TJ integral membrane protein; *EB1* end-binding 1 protein, a microtubule plus (+)-end tracking protein, or +TIP; *Eps8* epidermal growth factor receptor pathway substrate 8, an actin barbed end capping and bundling protein; *ZO-1* zonula occludens-1.

### Preparation of cDNA constructs and cloning into pCI-neo mammalian expression vector

Different cDNA constructs for laminin-type globular (LG) domains of LG3, LG4, LG5, LG3/4 and LG4/5 were obtained by PCR using corresponding primer pairs specific to LG3, LG4 or LG5 based on Genbank Accession Number XM_017590489.1 for rat laminin-α2 chain (Table 1) with cDNAs reverse-transcribed from Sertoli cell RNAs to serve as the PCR template. For LG3/4 or LG4/5, the sense primer of LG3 or LG4 and the antisense primer of LG4 or LG5 were used, respectively, for PCR (Table 1). For LG3/4/5 (80 kDa laminin-α2 chain fragment), the PCR products from LG3/4 and LG4/5 were heat denatured, re-annealed, and serve as the template for PCR by using prime pair of LG3 (sense) and LG5 (antisense). The corresponding cDNA constructs obtained by PCR with the expected size in bp were subjected to direct nucleotide sequencing to confirm their identity at Genewiz (South Plainfield, NJ). These cDNA constructs were then cloned into the pCI-neo vector (Promega) at the *Xho*I/*Sal*I restriction sites. Plasmid DNA was purified using ZymoPURE^TM^11 Plasmid Midiprep Kit (Zymo Research, Irvine, CA) which also removed possible endotoxin contamination to a negligible level. Plasmid DNA was then labeled with Cy3 using a Label*IT* Tracker Intracellular Nucleic Acid Localization kit (Mirus) to track successful transfection.

### Overexpression of cDNAs in primary cultures of Sertoli cells

Sertoli cells were isolated from testes of male pups at 20 days of age and cultured in F12/DMEM serum-free medium (Sigma-Aldrich) as described^20^. In brief, freshly isolated Sertoli cells were seeded on Matrigel (Fisher Scientific)-coated culture dishes (either 6-, 12- or 24-wells), round coverslips (18-mm diameter, to be placed in 12-well dishes) and bicameral units (Millipore Millicell-HA culture inserts, 12-mm in diameter; Millipore, Billerica, MA) (to be placed in 24-well dishes) at a density 0.4–0.6×, 0.025–0.04×, and 1 × 10^6^ cells/cm^2^, respectively^20^. For 6-, 12- or 24-well dishes, each well contained 5-, 2- or 1-ml F12/DMEM medium, supplemented with growth factors (bovine insulin, human transferrin, EGF), bacitracin and gentamicin^20^, which were used for either IB or nucleic acid extraction. For cells cultured on coverslips to be used for IF (placed in 12-well dishes), each well contained 2-ml F12/DMEM. For bicameral units, which were placed in 24-well dishes, the apical and basal compartment contained 0.5-ml each of F12/DMEM. All Sertoli cell-containing culture dishes were then placed a humidified CO_2_-incubator with 95% air/5% CO_2_ (vol/vol) at 35 °C. In these primary Sertoli cell cultures, ultrastructures of actin-based TJ, basal ES and gap junction, as well as intermediate filament-based desmosome that mimicked the Sertoli cell blood–testis barrier (BTB) in vivo were detected when examined by electron microscopy^21–23^. It was noted that our Sertoli cell cultures were almost 98% pure with minimal Leydig, germ and peritubular myoid cell contaminations based on RT-PCR using primer pairs specific to Leydig, germ, and peritubular myoid cell markers as described^24^. Sertoli cells were used for transfection experiments on day 3 after a functional tight junction (TJ)-permeability barrier was established. This experiment was used to assess if overexpression of any of LG cDNAs would block the cadmium-induced Sertoli cell TJ-permeability barrier function, protein distribution, and F-actin or MT-organization as follows. In brief, Sertoli cells were treated with CdCl_2_ (1 µM) for 6-h. Thereafter, cells were rinsed thrice to remove the toxicant, and transfected on day 4 with different LG cDNAs, namely LG3, LG4, LG5, LG3/4, LG4/5, and LG3/4/5 vs. empty vector (i.e., pCI-neo/Ctrl), using the corresponding plasmid DNA (using 0.45 μg plasmid DNA per 10^6^ Sertoli cells) for 6 h using Lipojet In Vitro Transfection Reagent (SignaGen Laboratories, Rockville, MD) with a 3-μl transfection medium: 1-μg plasmid DNA ratio as earlier described^5^. Thereafter, transfection reagent was removed and cells were rinsed with F12/DMEM (thrice), and incubated with F12/DMEM. For cultures to be used for IF, plasmid DNAs were labeled with Cy3-based Label*IT*® Tracker™ Intracellular Nucleic Acid Localization Kit (Mirus Bio, Madison, WI) to track successful transfection^25^. Cells were harvested 2 days thereafter for fluorescence microscopy and/or preparation of lysates for IB. TER was measured once daily throughout the experimental period to assess any changes in Sertoli cell TJ-permeability barrier function. For all in vitro experiments, data were representative findings from an experiment of *n* = 3 independent experiments using different batches of Sertoli cells which yielded similar observations. Each treatment vs. control groups had at least triplicate wells.

### Overexpression of cDNAs in rat testes in vivo to assess the protective effects of LG5- and LG3/4/5-peptides on cadmium-induced testis injury

We examined the protective effects of LG5- and LG3/4/5-peptides on cadmium-induced testis injury using two approaches. (a) Assessing the effects of LG5- and LG3/4/5-peptides to block CdCl_*2*_-induced testis injury. In this approach, testes were transfected with pCI-neo/LG3/4/5 vs. pCI-neo/LG5 thrice on day 0, 2, and 4, to be followed by CdCl_2_ treatment on day 6 (3 mg/kg b.w. via i.p. using a stock solution of 10% CdCl_2_ in sterile PBS (wt/vol)) which is known to induce BTB disruption^26–28^. pCI-neo/LG5 was selected for comparison since in vitro studies illustrated that LG5 was the fragment containing the biologically active domain, capable of blocking Cd-induced Sertoli cell TJ-barrier disruption. Following treatments, the BTB integrity in vivo was assessed. In brief, each testis was transfected with 15 µg plasmid DNA (either pCI-neo/LG3/4/5 or pCI-neo/LG5) together with 1.8 µl in vivo-jetPEI reagent (PolyPlus-transfection, Illkirch-Graffenstaden, France), suspended in sterile 5% glucose (wt/vol) to a final transfection mixture of 70 µl per testis. In brief, one testis (at ~1.6 gm per testis with a volume of ~1.6 ml) of the same rat received 70 µl of transfection mixture containing empty pCI-neo vector DNA (control) vs. the other testis received the same volume of transfection mixture containing either pCI-neo/LG3/4/5 or pCI-neo/LG5 plasmid DNA, which was administered to each testis via a 28-gauge insulin syringe. In order to avoid an acute rise in intra-testicular hydrostatic pressure during transfection mixture administration, needle was inserted vertically from the apical to the basal end of the testis, and as the needle was withdrawn, transfection solution was gently released and filled the entire testis as described^15,29^. Using this approach, the transfection efficiency was shown to be ~70% as earlier reported^15,29^. Thereafter, rats were euthanized on day 7.5 by CO_2_ asphyxiation using slow (20–30%/min) displacement of chamber air from a carbon dioxide tank. Testes were removed immediately, frozen in liquid nitrogen or fixed in Bouin’s fixative or modified Davidson’s fixative^30,31^ for either histological analysis or to be used to assess the BTB integrity in vivo. This approach thus examined if LG3/4/5 or LG5 vs. pCI-neo/Ctrl overexpression blocked the cadmium-induced testis injury. (b) Assessing the effects of LG3/4/5 to rescue testis injury induced by CdCl_2_. In this second approach, treatment of rats with CdCl_2_ was performed on day 0, to be followed by transfecting the testis with pCI-neo/LG3/4/5 for overexpression on day 1, 2,and 3 (thrice). Thereafter, rats were euthanized on day 56 for either histological analysis (following fixation and paraffin embedding, see above) or to assess the BTB integrity, thereby assessing if LG3/4/5 was able to rescue testes from cadmium-induced injury. Each treatment group including controls containing *n* = 4 rats in these two studies, excluding animals used for pilot experiments with *n* = 2 rats for each treatment and control groups which were used to assess the optimal conditions to assess changes in phenotypes. Investigators in this study were not blinded to the group allocation during the experiment, and when assessing the outcome.

### Transfection efficiency of different cDNA constructs in the testis in vivo

Transfection efficiency of cDNA constructs in the testis in vivo was estimated as earlier described^15^. In brief, testes were transfected by pCI-neo plasmid DNA cloned with a *Discosoma sp*. red fluorescence protein DsRed2 (i.e., pCI-neo/DsRed2) of 678 bp (see ref. ^15^) using procedures as described above. In selected experiments, overexpression of a target gene such as LG3/4/5 in the testis in vivo was also performed together with pCI-neo/DsRed2 (i.e., co-transfection) to estimate the transfection efficiency as described^15,32^. Fluorescence aggregates of pCI-neo/DsRed2 was randomly scored in ~80 tubules per testis, using cross-sections of testes from *n* = 3 rats on 7.5 day (see regimen used to investigate the blocking effects of LG3/4/5 on cadmium-induced injury in the testis) in both experiments, namely pCI-neo/DsRed2 (single transfection) or co-transfection with pCI-neo/DsRed2 and pCI-neo/LG3/4/5 (dual transfection). Successful transfection was confirmed by scoring the presence of at least 10 aggregates of red fluorescence in the cross-section of a randomly tubule from rat testes. Using this approach, transfection efficiency was estimated to be ~70% after 240 cross-sections of seminiferous tubules were randomly scored from *n* = 3 rats using PolyPlus in vivo-jetPEI® as the transfection reagent for the single or dual transfection group, and findings from this study were consistent with earlier reports from our laboratory^15,29,32^.

### Monitoring Sertoli cell TJ-permeability barrier function in vitro and BTB integrity in vivo

(a) In vitro Sertoli cell TJ-barrier functional assay: the TJ-permeability barrier function across the Sertoli cell epithelium was assessed by using Sertoli cells cultured on bicameral units (diameter 12 mm; pore size 0.45 µm, effective surface area 0.6 cm^2^; EMD Millipore) at 1.0 × 10^6^ cells/cm^2^ as described^20^. In brief, each bicameral unit was placed inside the well of a 24-well dish with 0.5 ml F12/DMEM each in the apical and the basal compartments. In order to monitor the promoting effects of LG3/4/5 on the BTB function, Sertoli cells cultured alone on day 3 with ultrastructures of TJ, basal ES, gap junction and desmosome^21,22^ were treated with CdCl_2_ (1 µM) for 6 h, which is known to induce reversible Sertoli cell TJ-permeability barrier disruption^33,34^. Thereafter, CdCl2 was removed by rinsing cells thrice with fresh F12/DMEM, and cells were transfected on day 4 with the corresponding plasmid DNA for 6 h for overexpression of LG3/4/5 and LG5 vs. pCI-neo (empty vector), and TJ-barrier function was monitored daily until day 7 by quantifying the transepithelial electrical resistance (TER) across the Sertoli cell epithelium as described^5,20^. For this study, each treatment and control group had quadruple replicates from *n* = 4 independent experiments using different batches of Sertoli cells which yielded similar results. (b) In vivo BTB integrity assay: the integrity of the BTB in the testis in vivo was monitored as earlier described^15,35^. This assay was based on the ability of a functional and intact Sertoli cell BTB that blocked the movement of a small membrane impermeable biotin reagent sulfo-NHS-LC-biotin with a Mr of 556.59 (Thermo Fisher Scientific, Waltham, MA) across the BTB. In brief, rats (*n* = 3 rats per treatment vs. control groups) were anesthetized by ketamine HCl (60 mg/kg b.w., i.m.) with xylazine (10 mg/kg b.w., i.m.) as an analgesic. Thereafter, 100 µl of 10 mg/ml sulfo-NHS-LC-biotin dissolved in PBS (10 mM sodium phosphate, 0.15 M NaCl, pH 7.4 at 22 °C) containing 1 mM CaCl_2_ was loaded gently under the tunica albuginea using a 28-gauge insulin needle. Thus, the biotinylation reagent diffused across a disrupted BTB when it was compromised by CdCl_2_ treatment, and readily detected in the adluminal compartment of the epithelium. After 30 min, rats were euthanized by CO_2_ asphyxiation, testes were immediately removed, snap-frozen in liquid nitrogen. Frozen sections (~10-µm thickness) were obtained in a cryostat (−22 °C), fixed in 4% PFA (paraformaldehyde, in PBS, wt/vol) for 10 min, to be followed by Alexa Fluor 488-streptavidin (1:250; green fluorescence) for 30 min. Slides were then mounted with Prolong Gold Antifade reagent with DAPI (Life Technologies). pCI-neo/Ctrl (empty vector) served as negative control group.

### RNA extraction and RT-PCR

Total RNA was isolated from rat testes or Sertoli cells using TRIZOL reagent (Life Technologies) for RT-PCR as described^5,35^. In brief, 2 µg total RNA was reverse transcribed by M-MLV (Moloney murine leukemia virus) reverse transcriptase (Promega, Madison, WI) to obtain total cDNAs which served as templates for subsequent PCR. PCR was performed using primer pairs specific to corresponding target genes vs. S16 which served as the PCR loading control (Table 1). Authenticity of PCR products was verified by direct DNA sequencing at Genewiz (South Plainfield, NJ). Each RT-PCR experiment had *n* = 3 experiments which yielded similar results. RT-PCR was also used to confirm specific overexpression of LG3/4/5 vs. LG5.

### Lysate preparation, protein estimation, immunoblot (IB) analysis, and chemiluminescence analysis

Lysates of testes or Sertoli cells were obtained using lysis buffer [50 mM Tris, containing 0.15 M NaCl, 1% Nonidet P-40 (vol/vol), 1 mM EGTA, 10% glycerol (vol/vol), pH 7.4 at 22 °C, freshly supplemented with protease inhibitor mixture (Sigma-Aldrich) and phosphatase inhibitor cocktail II (Sigma-Aldrich)] for immunoblot analysis^36^. Protein estimation was performed using BSA as a standard with a Bio-Rad DC protein assay kit (Bio-Rad Laboratories, Hercules, CA). Immunoblot analysis was performed using equal amount of total protein lysate between samples in each experiment at 40 μg protein per lane. Corresponding antibodies specific to each target protein were listed in Table 2 using in-house prepared chemiluminescence kits^20^. Chemiluminescence signals of protein blots were detected using an ImageQuant LAS 4000 mini (GE Healthcare Life Sciences) Imaging system and ImageQuant software (Version 1.3). β-actin or α-tubulin served as a protein loading control. Protein blots were quantified by ImageJ 1.45 s software obtained at http://rsbweb.nih.gov/ij (National Institutes of Health (NIH), Bethesda, MD). All samples within an experimental group were processed simultaneously to avoid inter-experimental variations. Each sample had triplcates in treatment vs. control groups from *n* = 3 independent experiments using different rat testes.

### Histology, immunofluorescence analysis, F-actin and α-tubulin staining, and fluorescence image analysis

Histology was performed using cross-sections of testes fixed in modified Davidson’s fixative, embedded in paraffin and cut with a microtome (5 µm in thickness) for H&E (hematoxylin and eosin) staining as described^37^. Immunofluorescence analysis (IF) was performed using frozen cross-sections of testes at 7 μm obtained in a cryostat at −22˚C, or Sertoli cells cultured on coverslips. These testis sections or Sertoli cells were fixed in PFA (4%, in PBS, wt//vol) as described^35,37^. Target proteins (including α-tubulin to visualize MTs) were visualized by incubating testis sections or cells with a specific primary and the corresponding secondary antibodies (Table 2). Cell nuclei were visualized by 4′,6-diamidino-2-phenylindole (DAPI) (Sigma). Slides were mounted in Prolong Gold Antifade reagent (Invitrogen, Life Technologies). For F-actin staining, sections or Sertoli cells were incubated with Alexa Fluor 488 phalloidin (Invitrogen) according to manufacturer’s instructions as described^35^. Images were examined and acquired using a Nikon Eclipse 90i Fluorescence Microscope system equipped with Nikon Ds-Qi1Mc or DsFi1 digital camera and Nikon NIS Elements AR 3.2 software (Nikon, Tokyo, Japan) in TIFF format. Image overlays were performed using Adobe Photoshop CS4 (San Jose, CA) to assess co-localization of proteins at the same cellular sites. Fluorescence intensity was analyzed by ImageJ 1.45 s (NIH, Bethesda, MD) or Nikon NIS Elements AR (Version 3.2) software package. All samples from any experiment including both treatment vs. control groups were analyzed simultaneously to avoid inter-experimental variations. Data shown here were representative data from a single experiment, with a total of *n* = 3 independent experiments which yielded similar results using different batches of Sertoli cells or rat testes. For fluorescence intensity or distribution analysis in Sertoli cells or seminiferous tubules from testes, at least 100 cells from a typical experiment or 100 cross-sections of each testis were randomly selected and examined in both experimental and control groups, and a total *n* = 3 experiments were performed.

### Statistical analysis

Statistical analysis was performed with GraphPad Prism 6 software (GraphPad Software) using either Student *t*-test (for two-group comparisons), one-way analysis of variance (ANOVA) (for multi-group comparisons), or two-way ANOVA with Bonferroni post hoc tests. All experiments had 3–5 replicate samples with a total of at least *n* = 3 experiments or testes from *n* = 3 rats to obtain enough data for meaningful statistical analysis.

## Results

### LG3/4/5 or LG5 promotes Sertoli cell TJ-barrier function by rescuing Cd-induced cell injury in vitro

**Fig. 1:**
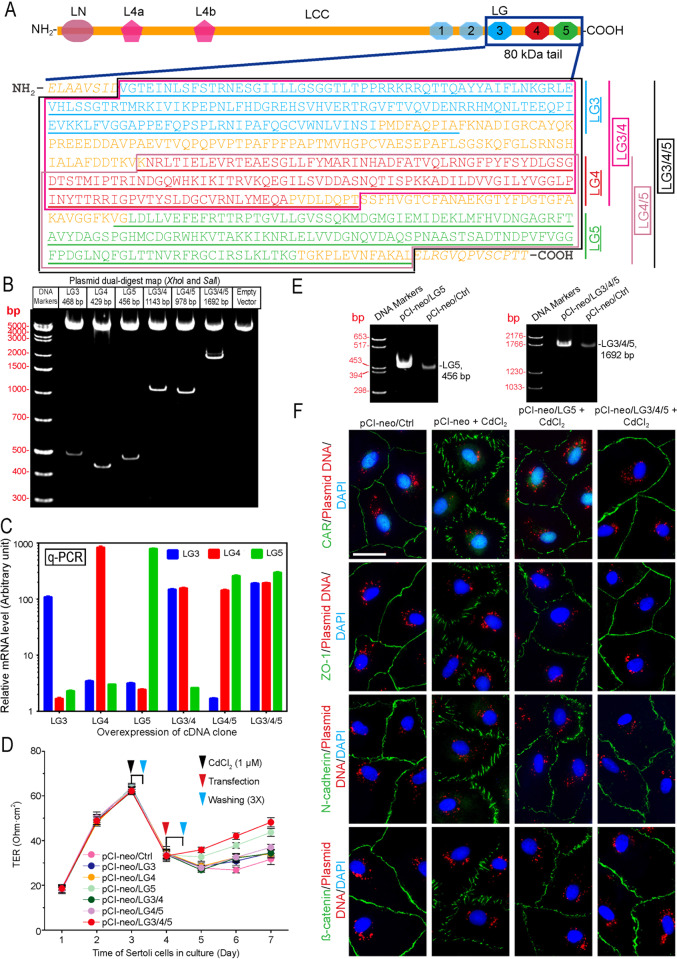
LG3/4/5 protects Sertoli cells from cadmium-induced TJ-barrier disruption. **a** Preparation of cDNA constructs based on the 80 kDa tail of the laminin-α2 chain. The top panel is a schematic drawing that illustrates the functional domains of laminin-α2 chain. From the N-terminus, the short arm of laminin-α2 is comprised of three globular domains: laminin N-terminal domain (LN), laminin 4a domain (L4a) and laminin 4b domain (L4b). The long arm of laminin-α2 is comprised of laminin coiled coil (LCC) domain and five C-terminal laminin-type globular (LG) domains of LG1, 2, 3, 4, and 5. Proteolytic cleavage is close to the N-terminus of LG3^67^ to generate the LG3/4/5 (i.e., the 80 kDa fragment). Six different cDNA constructs, namely LG3 (underlined in blue), LG4 (underlined in red), LG5 (underlined in green), LG3/4 (boxed in pink), LG4/5 (boxed in purple), and LG3/4/5 (boxed in black), were obtained by PCR using Sertoli cell cDNAs as the template and the corresponding specific primer pairs (Table 1), with the exception of the LG3/4/5 clone which was obtained by first denaturing LG3/4 and LG4/5, so that these two clones were annealed and then served as the template for PCR by using primer pairs (1) and (6). These constructs were cloned into the mammalian expression vector pCI-neo (Promega). Amino acid residues in orange represent sequences between the different LG domains and the italicized orange residues were not cloned into any of the cDNA constructs, such as LG3, LG4 or LG5. However, LG3/4, LG4/5, and LG3/4/5 contained the linker sequences noted in orange (but not the italicized orange sequences). All constructs were subsequently confirmed by directly nucleotide sequencing in Genewiz (South Plainfield, NJ). **b** cDNAs were obtained with their identity confirmed by nucleotide sequence analysis at Genewiz. Each fragment incorporates *Xho*I and *Sal*I restriction sites at its 5′- and 3′-end, respectively, for cloning. Orange colored residues are linker sequences between the LG3, LG4 and LG5 domains, the italicized orange colored residues were not cloned into the cDNA constructs. **c** Following overexpression in primary Sertoli cell cultures, the specificity of overexpression was confirmed by qPCR^68^. **d** Overexpression of LG5 and LG3/4/5, but not pCI-neo empty vector (Ctrl), in Sertoli cells was found to promote TJ-barrier function. Representation data of an experiment with triplicate bicameral units, and a total of *n* = 3 experiments that yielded similar results. *P < 0.05; **P < 0.01; Student’s *t*-test compared between corresponding treatment and pCI-neo control groups. **e** Overexpression of LG5 and LG3/4/5 was confirmed by RT-PCR for the experiment noted in **f** to examine their promoting effects on Sertoli cell epithelium to protect Sertoli cells from cadmium-mediated cell injury regarding disruptive changes on TJ (e.g., CAR, ZO-1) and basal ES (N-cadherin, ß-catenin) protein distribution at the Sertoli cell-cell interface. Results of representation experiment from *n* = 3 independent experiments. Scale bar, 40 µm, applies to all other micrographs.

LG3/4/5 cDNA, obtained by PCR using Sertoli cell total RNAs derived from laminin-α2 chain with the corresponding primer pairs (11 and 12, see Table 1), was cloned into the mammalian expression vector pCI-neo (Promega), containing the appropriate restriction sites (Table 1, Fig. 1). As noted in Fig. 1a, different fragments of LG3/4/5, which included LG3, LG4, LG5, LG3/4, LG4/5 vs. LG3/4/5 (note: some of these clones also contained the linker sequence) were cloned using the corresponding primer pairs (see Table 1). cDNAs (in bp) for different clones as noted in Fig. 1b, c, and their identity was confirmed by direct nucleotide sequencing at Genewiz (South Plainfield, NJ). Overexpression of either L5 or LG3/4/5, but not other cDNA clones, was found to promote Sertoli cell TJ-permeability barrier, capable of rescuing the cadmium-induced Sertoli cell injury regarding TJ-barrier function (Fig. 1d). Besides qPCR (Fig. 1c), overexpression of LG5 vs. LG3/4/5 was confirmed by RT-PCR (Fig. 1e) with S16 as the PCR control. Overexpression of the corresponding cDNA constructs, namely LG3, LG4, LG3/4, and LG4/5, in Sertoli cells was also confirmed, similar to findings noted in Fig. 1e, and with the corresponding bp sizes noted in Fig. 1b. As noted in Fig. 1f, CdCl_2_ exerted its disruptive effects by perturbing Sertoli cell TJ-barrier function through mis-localization of the TJ (e.g., CAR, ZO-1) and basal ES (e.g., N-cadherin, ß-catenin) proteins at the Sertoli cell cortical zone. Interestingly, overexpression of either LG5 or LG3/4/5 was capable of rescuing cadmium-induced Sertoli cell injury by correcting the disruptive distribution of the TJ and basal ES proteins as noted in Fig. 1f.

### LG3/4/5 or LG5 rescues cadmium-induced Sertoli cell injury by promoting cytoskeletal organization in vitro

**Fig. 2:**
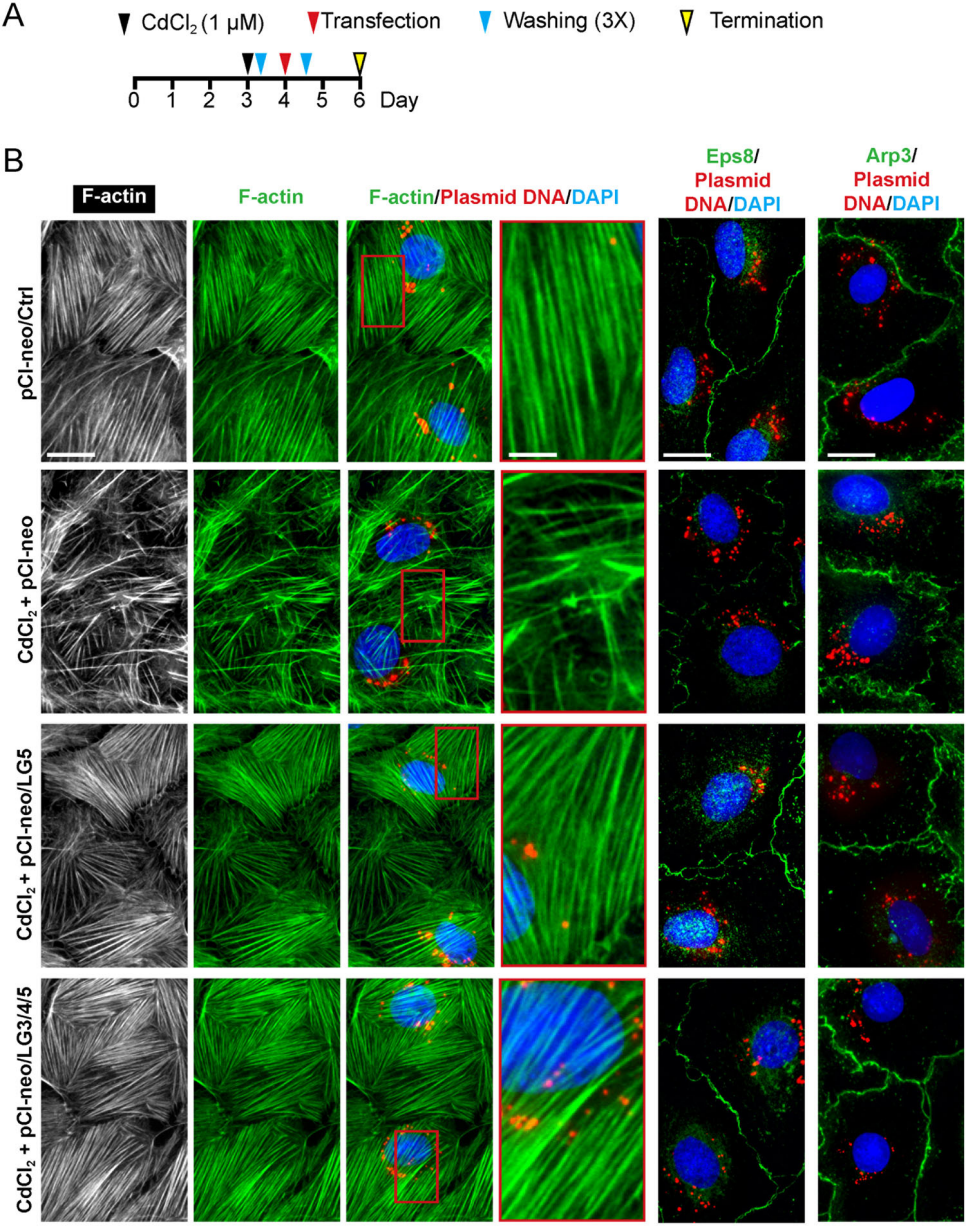
LG5 or LG3/4/5 blocks cadmium-induced Sertoli cell injury through changes in cytoskeletal organization of F-actin. **a** Regimen used for the study from *n* = 3 independent experiments using different batches of Sertoli cells which yielded similar results. **b** In control Sertoli cells transfected with vector empty (pCI-neo/Ctrl), actin filaments stretched across the Sertoli cells as distinctive linear filaments, and this typical organization of F-actin across the cell cytosol was maintained by typical spatial expression of actin barbed end capping and bundling protein Eps8 (promoted actin filaments to assume a bundled/linear configuration) and also branched actin polymerization protein Arp3 (which together with Arp2 that creates the Arp2/3 complex that promotes actin filaments to assume a branched configuration), which prominently localized at the Sertoli cell-cell interface. Following treatment with CdCl_2_ (without overexpression of either LG5 or LG3/4/5), actin filaments were grossly truncated (see inset of the corresponding magnified area boxed in red), possibly due to rapid internalization of Arp3, facilitating the generation of a branched actin network that de-stabilized Sertoli cell TJ-permeability function as noted in Fig. 1d through mis-distribution of TJ- and basal ES-proteins at the cell-cell interface (Fig. 1e), which utilized actin for their attachments. Importantly, overexpression of LG5 or LG3/4/5 considerably blocked the Cd-induced gross disruption of actin filaments across the cell cytosol, and this protective effect appeared to be more prominent in cells overexpressed with LG3/4/5 when the distributions of Eps8 and Arp3 were assessed. Plasmid DNA was labeled with Cy3 to track successful transfection. Data shown herein are representative micrographs of an experiment from *n* = 3 independent experiments which yielded similar results. Scale bar, 40 µm; inset, 20 µm; which apply to corresponding micrographs or insets.

**Fig. 3:**
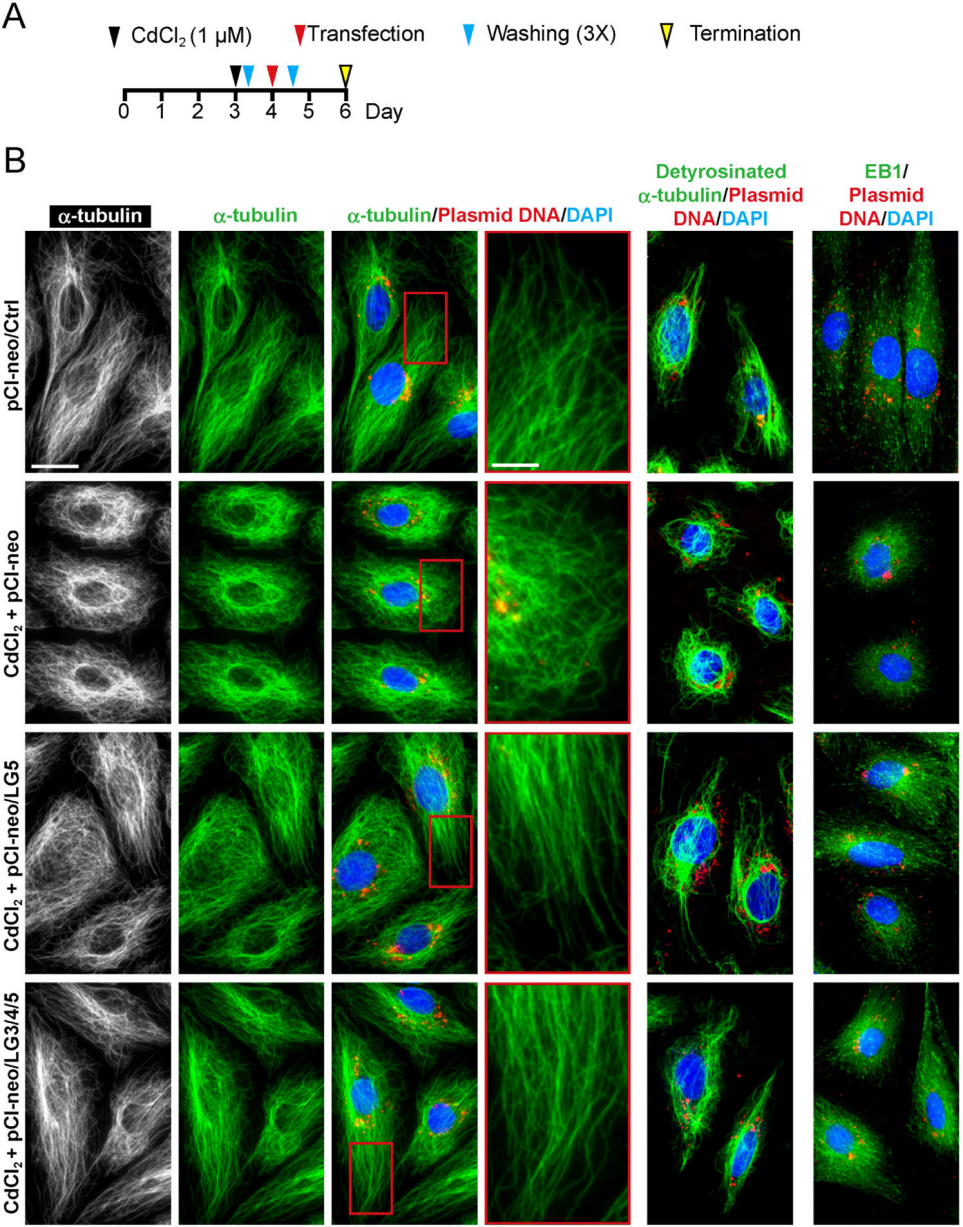
LG3/4/5 rescues cadmium-induced Sertoli cell injury through its promoting effects on MT cytoskeletal organization. The regimen used in this study that assessed the ability of LG5 or LG3/4/5 vs. pCI-neo/Ctrl (empty vector) to rescue Sertoli cells from CdCl_2_-induced injury is shown in Fig. 3a. In control cells, MTs (visualized by α-tubulin staining, which together with ß-tubulins create the α-/ß-tubulin dimers are the building blocks of MTs^69,70^) stretched across the Sertoli cell cytosol (see also magnified image boxed in red in inset). However, treatment of these Sertoli cells with CdCl_2_ at 1 µM for 6 h caused retraction of MTs from the cell peripheries, rendering MTs to stay closer to the cell nuclei, but also appeared as a randomly aligned branched network. Overexpression of LG5 or LG3/4/5 was found to block the cadmium-induced MT disorganization, supporting the notion that LG/5 and LG3/4/5 possessed the biological effects to promote BTB and spermatogenic functions by correcting the cadmium-induced MT-based cytoskeletal disorganization. On the two right panels, CdCl_2_ was also found to induce disruptive changes on the spatial expression of EB1 (a +TIP known to confer MT stability^41,71^) and detyrosinated α-tubulin (also known to confer MT stability^40^) since EB1 and detyrosinated α-tubulin no longer stretched across the Sertoli cell cytosol to support MTs, but retracted closer to the cell nuclei following CdCl_2_ treatment. But overexpression of LG5 or LG3/4/5 was capable of rescuing cadmium-induced disruptive distribution of either EB1 or detyrosinated α-tubulin across Sertoli cell cytosol to confer Sertoli cell function. Plasmid DNA was labeled with Cy3 (red fluorescence) to assess successful transfection. Data shown herein are representative micrographs of an experiment from *n* = 3 independent experiments which yielded similar results. Scale bar, 40 µm; inset, 20 µm; which apply to corresponding micrographs or insets.

Figure 2a shows the regimen used for the study to assess the effects of LG5 and LG3/4/5 to rescue cadmium-induced Sertoli cell injury (Figs. 2b and 3). In controls, F-actin stretched across the Sertoli cell cytosol as linear filaments to confer cell shape and maintained TJ-barrier function, which in turn supported by the concerted spatial expression of barbed end capping and bundling protein Eps8^38^ and branched actin polymerization protein Arp3^39^ (Fig. 2b). Treatment of Sertoli cell epithelium with a functional TJ-barrier (Fig. 1d) with CdCl_2_ perturbed F-actin organization by causing extensive defragmentation of actin filaments and branching due to disruptive changes in spatial expression of Eps8 and Arp3 (Fig. 2b, second row vs. top row). However, overexpression either LG5 or LG3/4/5 was able to rescue the CdCl_2_-induced Sertoli cell injury by correcting the cadmium-mediated cytoskeletal disorganization of F-actin (Fig. 2b), supporting the notion that the active domain in LG3/4/5 resides in LG5-peptide. Similarly, MTs (visualized by staining of α-tubulin, which together with ß-tubulin create the α-/ß-tubulin dimeric building blocks of MTs) stretched across the entire Sertoli cell cytosol as linear filaments in control Sertoli cells (Fig. 3). MTs in control cells were supported by detyrosinated α-tubulin, that is, removal of C-terminal Tyr by exposing Glu at the newly formed C-terminus, thereby stabilizing MTs by making them less dynamic^40^ wherein α-tubulin also stretched as linear fragments across cell cytosol (Fig. 3). Also, EB1 (end binding protein 1, a MT plus (+) end tracking protein, +TIP) known to stabilize MTs appeared as dot-like structures by binding to the plus ends of MTs that stretched across the cell cytosol^41^ as noted in control Sertoli cells (Fig. 3). However, CdCl_2_ induced extensive branching of MTs wherein MT no longer stretched across the cell cytosol as noted in control cells, but wrapped around the cell nuclei, including the detyrosinated α-tubulin and EB1 (Fig. 3). However, overexpression of either LG5 or LG3/4/5 was effective to rescue the CdCl_2_-induced cytoskeletal organization of MTs by correcting the proper distribution of detyrosinated α-tubulin and EB1 (Fig. 3).

### LG5 or LG3/4/5 overexpression blocks cadmium-induced testis injury in vivo

**Fig. 4:**
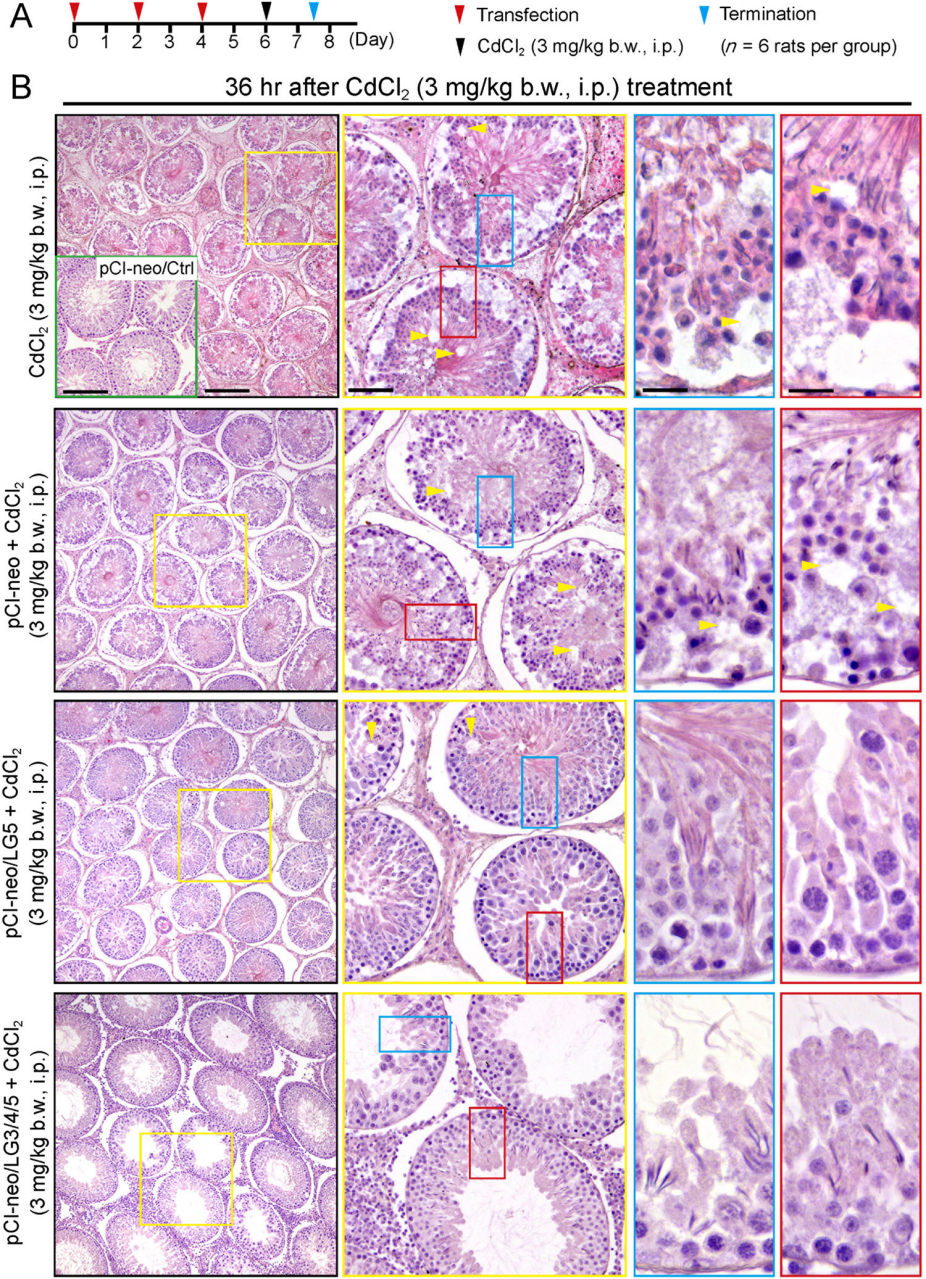
LG5- or LG3/4/5-peptide protects testes from cadmium-induced injury in vivo. **a** Regimen used in this experiment to assess the protective effects of LG5 or LG3/4/5 on cadmium-induced testis injury. In brief, groups of rats, *n* = 3 rats per treatment *vs*. control groups (i.e., rats transfected with pCI-neo (empty vector) without CdCl_2_ as noted in inset boxed in green) were used. Four treatment groups include: (i) CdCl_2_ alone, (ii) pCI-neo + CdCl_2_, (iii) pCI-neo/LG5 + CdCl_2_, and (iv) pCI-neo/LG3/4/5 + CdCl_2_. CdCl_2_ treatment was the use of CdCl_2_ at 3 mg/kg b.w., i.p., which was earlier shown to induce irreversible BTB disruption^26,28,45^ in rats. Results shown herein were derived from two independent experiments with *n* = 3 rats for all treatment and control groups for each experiment, which yielded similar results. **b** 36 hr after CdCl_2_ treatment, rats were euthanized. Thereafter, testes were immediately removed, fixed in modified Davidson’s fixative, embedded in paraffin and 5 µm cross-sections (thickness) using a microtome, stained with hematoxylin and eosin as described^37^ for histological analysis. Treatment of rats with CdCl_2_ without or with transfection with pCI-neo (empty vector) led to extensive seminiferous epithelial damage, manifested by massive germ cell exfoliation, Sertoli cell injury (noted by formation of large empty vacuoles in the epithelium annotated by yellow arrowheads), and general epithelial disorganization as noted in the magnified images boxed in blue or red. However, overexpression of LG5 of laminin-α2 was found to block cadmium-induced testis injury, and the effects of LG3/4/5 were even more remarkable since >90% of the tubules examined had displayed relatively normal spermatogenesis without noticeable defects as shown herein. These figures are also consistent with the in vitro data shown in Fig. 1d when the protective effects of LG3/4/5 vs. LG5 and other fragments were compared regarding their effects to protect Sertoli cell TJ-barrier function from CdCl_2_-induced barrier disruption. Scale bar, 400 µm, which applies to pCI-neo/Ctrl (inset) and also micrograph of the first row; 160 µm in micrograph in second row; 80 µm in insets in blue or red; which apply to corresponding micrographs or insets.

We next performed two in vivo studies to examine the promoting effects of LG3/4/5 and LG5 on spermatogenesis in the testis with a transfection efficiency of ~70% when estimated by using a DsRed2 red fluorescence protein and Polyplus in vivo-jetPEI® transfection medium. Findings from the first study were shown in Fig. 4 by investigating if LG3/4/5 vs. LG5 was effective in blocking CdCl_2_-induced testis injury using the regimen shown in Fig. 4a with *n* = 4 rats for each of the four groups including the pCI-neo/Ctrl (Group 5, see inset on the left micrograph, first row). Transfection of rat testes with pCI-neo (empty vector) alone had no effects on the status of spermatogenesis, consistent with earlier reports^15^, and similar to normal rat testes also noted in our pilot experiments (see inset in the first micrograph in the top panel) (Fig. 4b). By 36 h after CdCl_2_ treatment, extensive epithelial damage in the testis was noted, both in low resolution and enlarged images. These damages included: (i) premature release of spermatids, round spermatids and spermatocytes from the epithelium with these germ cells filled up the tubule lumen, (ii) extensive vacuoles were noted in the epithelium due to Sertoli cell injury and germ cell loss (annotated by yellow arrowheads), and (iii) shrinkage of the tubule diameter by at least 25% within 36 h after CdCl_2_ treatment (Fig. 4b, compared to the first two panels of CdCl_2_ treated groups), consistent with earlier reports regarding cadmium-induced testis injury^26–28,42^. However, overexpression of LG5 or LG3/4/5 as noted in regimen shown in Fig. 4a, LG5 and LG3/4/5 were capable of blocking CdCl_2_-induced testis injury since epithelial damage was considerably blocked in the pCI-neo/LG5 group (Fig. 4b). Furthermore, overexpression of LG3/4/5 appeared to be more effective in blocking the CdCl_2_-induced testis injury, consistent with findings shown in Fig. 1d. For instance, many of the tubule lumen remained filled with germ cells but spermatogenesis progressed well in the seminiferous epithelium in the pCI-neo/LG5 group (Fig. 4b), but the protective effects of LG3/4/5 in blocking CdCl_2_-induced testis injury regarding the status of spermatogenesis were remarkable (see last panel vs. other groups in Fig. 4b).

### Overexpression of LG3/4/5 rescues cadmium-induced testis injury in vivo

**Fig. 5:**
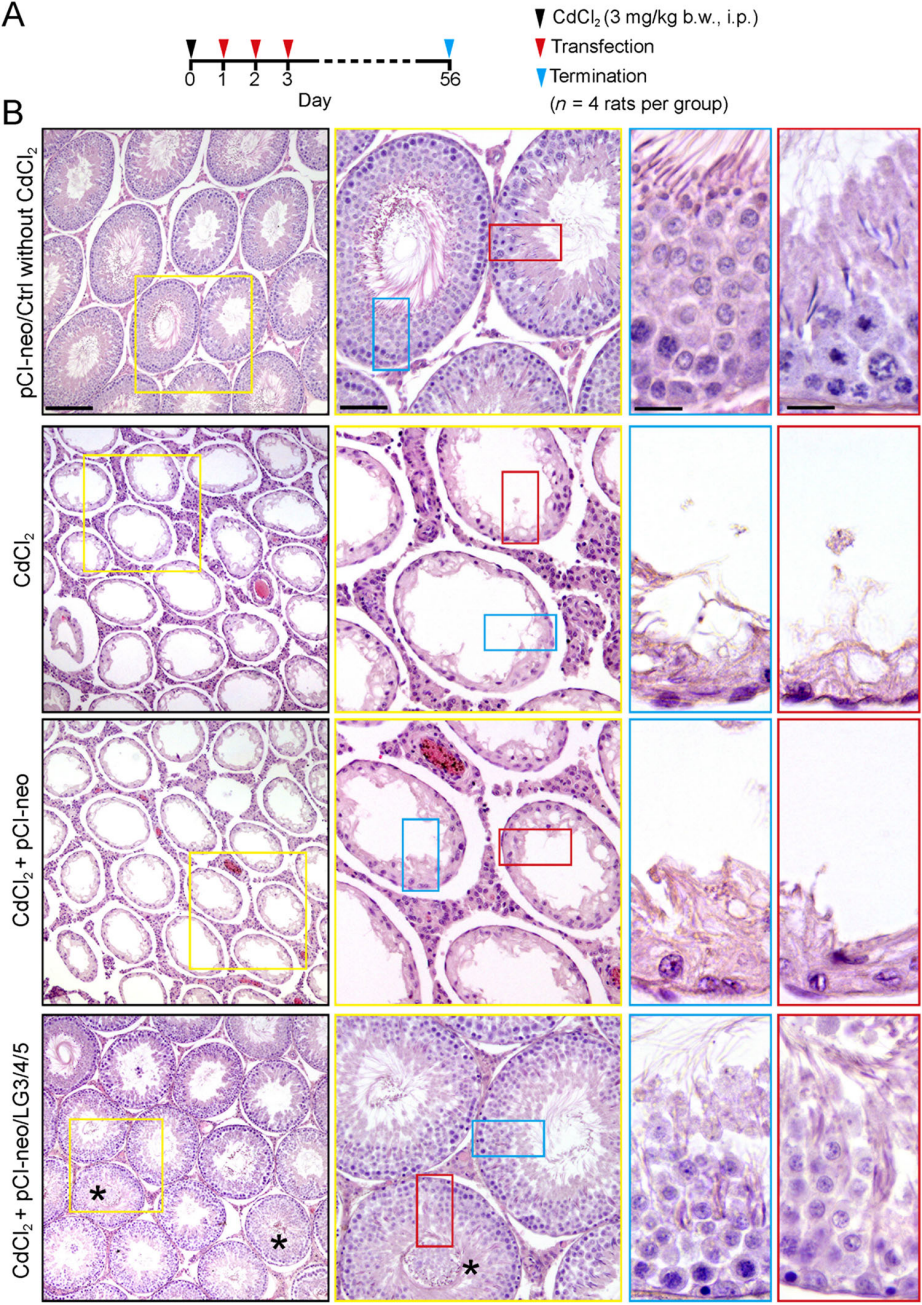
LG3/4/5 rescues testes from cadmium-induced injury in vivo. **a** Regimen used in this study to assess the protective effects of LG3/4/5 to rescue testes from cadmium-induced injury. Rats were treated with a single dose of CdCl_2_ on day 0, to be followed by three consecutive transfection of rats with pCI-neo/LG3/4/5 daily (thrice), and rats were terminated on day 56. In brief, 4 groups of rats (*n* = 4 rats per group) were used: (i) control rats transfected with empty vector alone (pCI-neo/Ctrl), (ii) CdCl_2_ alone (3 mg/kg b.w., i.p.), (iii) CdCl_2_ + pCI-neo, and (iv) CdCl_2_ + pCI-neo/LG3/4/5. **b** In control testes transfected with empty vector (pCI-neo/Ctrl), the status of spermatogenesis was normal and unaffected. However, either CdCl_2_ alone or transfected with pCI-neo on 2nd and 3^rd^ rows, considerably epithelial damage in the testis was noted wherein virtually 98% of the tubules were devoid of germ cells except for undifferentiated germ cells and Sertoli cells. However, transfection of the testis with LG3/4/5 (thrice) after CdCl_2_ treatment was capable of blocking cadmium-induced testis injury considerably since only ~10–15% of the tubules had signed of defects of spermatogenesis, with the phenotype most predominantly noted was premature release of spermatids into the tubule lumen in non-stage VIII tubules as shown herein (see 3rd and 4th rows in the last column vs. other panels). Scale bar, 400 µm in pCI-neo/Ctrl, 160 µm in the magnified micrograph boxed in yellow, 80 µm in insets in blue or red, which apply to corresponding micrographs or insets.

To further investigate the spermatogenesis promoting effects of LG3/4/5, we used another cadmium-based animal model with the regimen shown in Fig. 5a by examining if LG3/4/5 rescued cadmium-induced testis injury (Fig. 5b). As noted in control group, wherein testes were transfected with pCI-neo empty vector alone (pCI-neo/Ctrl) without CdCl_2_ treatment, the status of spermatogenesis across the entire epithelium was normal, similar to normal rat testes. However, a single dose of CdCl_2_ (3 mg/kg b.w., i.p.) was capable of inducing extensive epithelial damage, similar to cadmium-induced epithelial damage as noted in Fig. 4b, with or without transfection with pCI-neo empty vector (Fig. 5b). However, overexpression of LG3/4/5 effectively rescued cadmium-induced testis injury, rendering the epithelial morphology similar to the control group without CdCl_2_ treatment in >90% of the tubules examined herein (Fig. 5b).

### LG3/4/5 (or LG5) blocks and rescues cadmium-induced testis injury in vivo through F-actin and MT re-organization

**Fig. 6:**
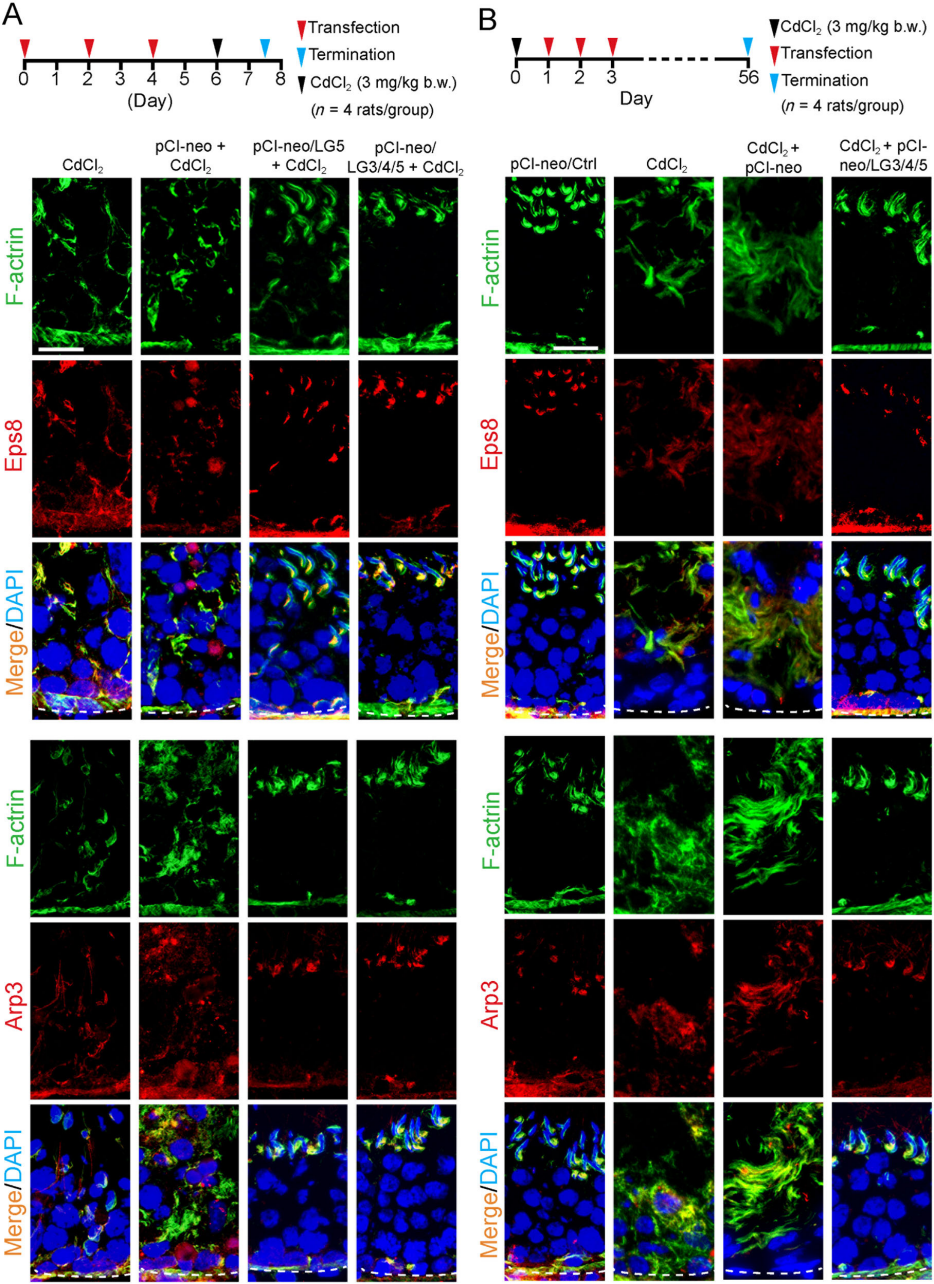
Protecting effects of LG5 and LG3/4/5 on spermatogenesis are mediated through changes in actin cytoskeletal organization. The two regimens used for the two experiments in **a**, **b** are shown on the top of the corresponding panels with *n* = 4 rats for each of the 4 groups for either experiment. Experiment shown in **a** was used to examine if overexpression of LG5 vs. LG3/4/5 blocked or **b** rescued cadmium-induced testis injury that led to disruptive organization of F-actin (green fluorescence). It was noted that overexpression of either LG5 or LG3/4/5 was effective in blocking cadmium-induced mis-localization of the two actin regulatory proteins Eps8 (red fluorescence, a barbed end capping and bundling protein that confers actin filaments to be assembled as bundles at the ES) and Arp3 (red fluorescence, a barbed end branched actin polymerization protein that converts linear actin filaments to a branched network) (**a**). LG3/4/5 was also effective in rescuing injury to testes induced by cadmium through disruptive spatial expression of Eps8 and Arp3 (**b**). Results are representative findings of an experiment, and *n* = 3 experiments using different testes yielded similar results. Scale bar, 80 µm, which applies to all other images in the same panel.

**Fig. 7:**
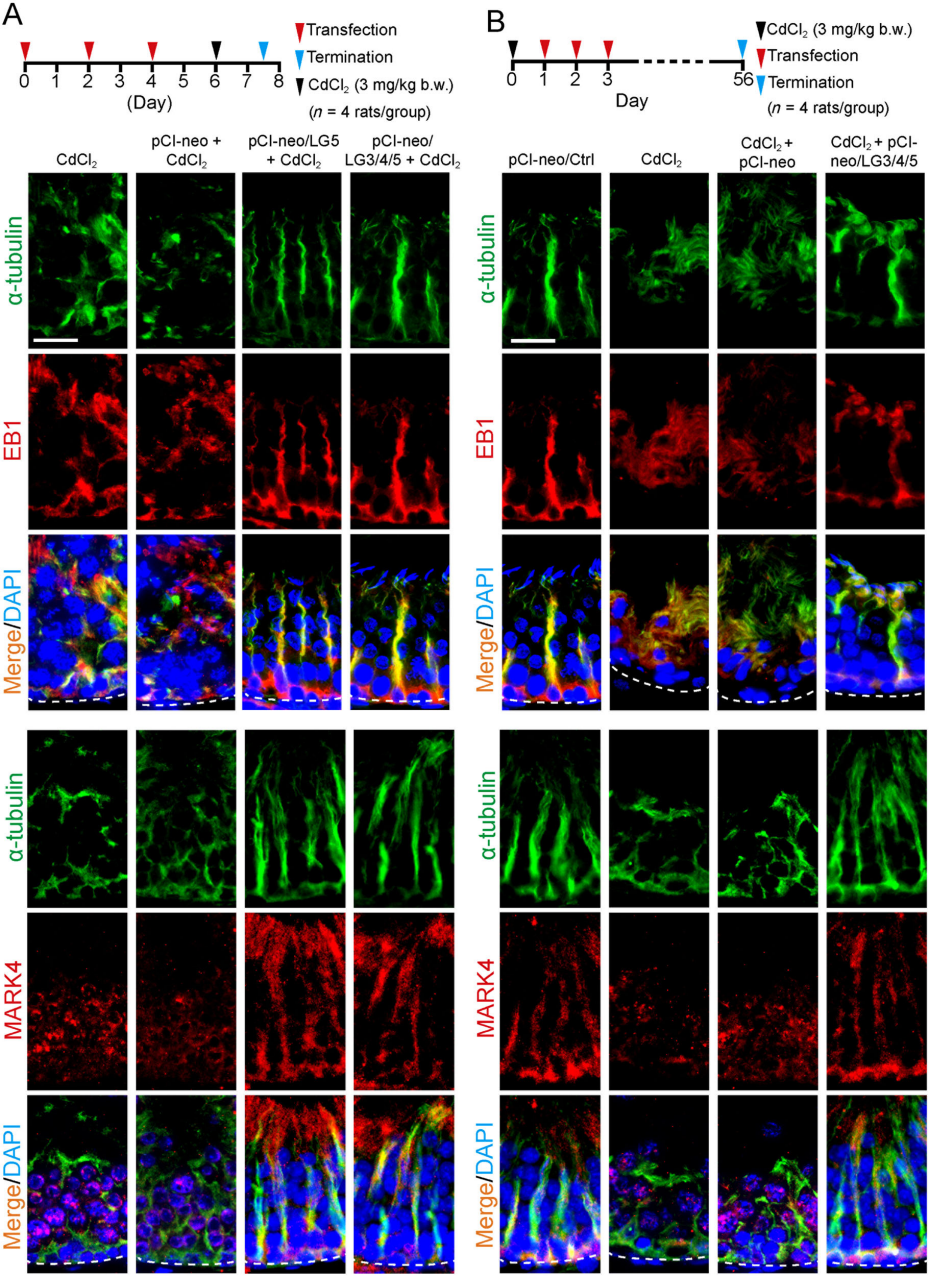
Protecting effects of LG5 and LG3/4/5 on spermatogenesis are mediated through changes in MT cytoskeletal organization. The two regimens used for the experiments in **a**, **b** are shown on the top of the corresponding panels with *n* = 4 rats for each of the 4 groups for either experiment. Experiment shown in **a** was used to examine if overexpression of LG5 vs. LG3/4/5 blocked or **b** rescued cadmium-induced testis injury that led to disruptive MT organization (visualized by α-tubulin, green fluorescence, which together with ß-tubulin that create the α-/ß-tubulin dimers, which serve as the building blocks of MTs). It was noted that overexpression of either LG5 or LG3/4/5 was effective in blocking **a**, **b** rescuing cadmium-induced cytoskeletal organization of MTs through mis-localization of the MT regulatory protein EB1 (red fluorescence, a +TIP which is known to stabilize MTs). Results are representative findings of an experiment, and *n* = 3 experiments using different testes yielded similar results. Scale bar, 80 µm, which applies to all other images in the same panel.

We investigated the underlying mechanism by which LG3/4/5 (or LG5) blocked (Fig. 6a) or rescued (Fig. 6b) cadmium-induced testis injury using the regimen noted in the top panel of Fig. 6a, b. Overexpression of LG5 or LG3/4/5 promoted the corrective spatial expression of the actin barbed end capping and bundling protein Eps8 and also the branched actin polymerization protein Arp3 to maintain F-actin cytoskeletal organization across the epithelium, thereby blocking testes from cadmium-induced injury (Fig. 6a). Similarly, using the regimen shown in the top panel of Fig. 6b, overexpression of LG3/4/5 rescued cadmium-induced testis injury by maintaining the spatial expression of the two F-actin regulatory proteins across the epithelium (Fig. 6b). On the other hand, CdCl_2_ also induced remarkable cytoskeletal disruption on the MT cytoskeleton as noted in Fig. 7a, b using the regimens shown in the top panel of either Fig. 7a, b by blocking or rescuing cadmium-induced MT disruption. For instance, as noted in the control group (pCI-neo/Ctrl) in Fig. 7b, MTs, visualized by staining with α-tubulin (green fluorescence, wherein α-tubulin that created the α-/ß-tubulin dimeric building blocks of MTs) appeared as tracks that laid perpendicular to the basement membrane (annotated by dashed white line at the base of the seminiferous tubule) (Fig. 7b). However, cadmium-induced remarkable damage to the MT cytoskeleton where MTs became truncated and extensive defragmented and by 56 days, MTs were virtually collapsed, no longer capable of supporting the seminiferous epithelium (Fig. 7a, b). However, overexpression of LG5 or LG3/4/5 was able to block cadmium-induced MT damage (Fig. 7a), and LG3/4/5 also rescued cadmium-mediated MT disruption (Fig. 7b). It appeared that LG3/4/5 or LG5 exerts their promoting effects through maintenance (or corrective expression) of the EB1 (a +TIP protein known to promote MT stabilization^41,43^) and MARK4 (also a regulator of MT dynamics^44^) (Fig. 7a, b).

### LG3/4/5 (or LG5) blocks or rescues cadmium-induced testis injury in vivo by maintaining BTB integrity

**Fig. 8:**
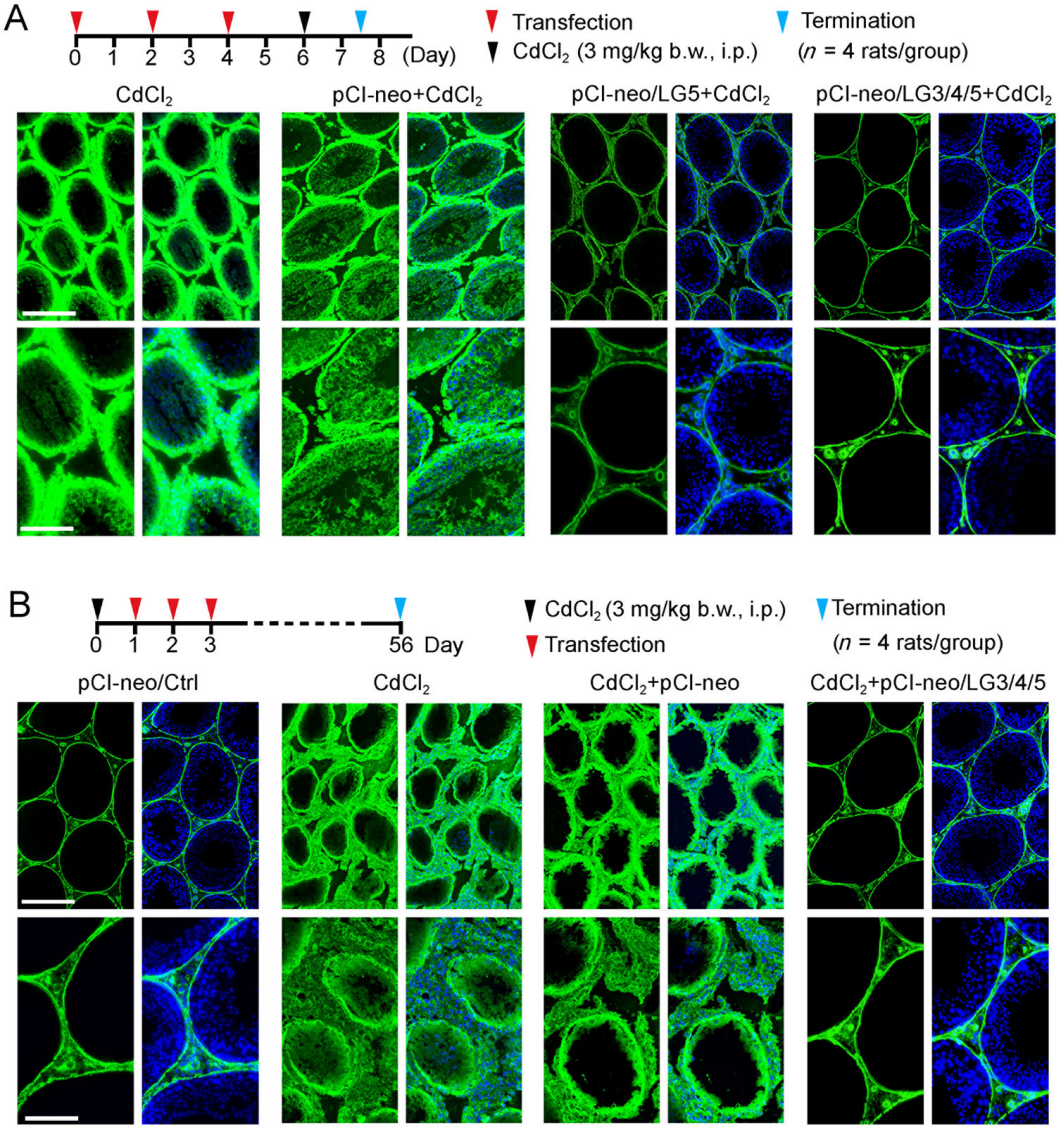
LG5 and LG3/4/5 protect the BTB against cadmium-induced testis injury in vivo. A study was performed to examine the promoting effects of LG5 vs. LG3/4/5 in blocking the cadmium-induced BTB disruption (**a**) and the promoting effects of LG3/4/5 in rescuing testes from cadmium-mediated BTB disruption (**b**). The regimen used for the experiment is in the top panel of the corresponding experiment with *n* = 4 rats per group. Results of the normal testes (−ve control) were not shown which were similar to the pCI-neo/Ctrl (another –ve control) as noted in **b**, wherein the intact BTB was capable of blocking the entry of a small membrane impermeable biotin (EZ-link sulfo-NHS-LC-biotin, Mr 556.59). Biotin was visualized by Alexa Fluor 488-streptravidin (green fluorescence). In rats treated with CdCl_2_, an environmental toxicant known to induce irreversible BTB disruption at this dose (3 mg/kg b.w., i.p.), biotin readily diffused into the seminiferous epithelium behind the BTB, covering virtually the entire epithelium in both CdCl_2_ and pCI-neo/CdCl_2_ group. However, overexpression of either LG5 or LG3/4/5 was capable of blocking cadmium-induced BTB disruption (**a**), and LG3/4/5 was also shown to rescue cadmium-mediated BTB disruption (**b**). Results shown herein are representative findings of an adult rat and *n* = 4 rats yielded similar results. Scale bar, 280 and 150 µm in the first and second panel in **a**, which also apply to **b** and the corresponding images in the same panel.

We next investigated the effects of LG5 vs. LG3/4/5 in blocking or LG3/4/5 in rescuing cadmium-induced testis injury through changes in BTB integrity via the use of an in vivo BTB integrity assay as shown in Fig. 8a, b using the corresponding regimen. It is known that CdCl_2_ exerts its toxic effects in the testis through its damaging effects on the BTB^26,27,45^, rendering BTB malfunctions, which in turn failing to support spermatogenesis^28^. In the pCI-neo/Ctrl group, the functional BTB was capable of blocking the entry of biotin (green fluorescence) into the seminiferous epithelium, but restricting biotin at the base of the tubule, unlike testes from rats treated with CdCl_2_ (3 mg/kg b.w., by i.p.; positive control known to induce irreversible BTB disruption^26,27^) wherein biotin was able to enter the seminiferous epithelium (see CdCl_2_ and pCI-neo/ClCl_2_ groups in Fig. 8a vs. pCI-neo/Ctrl in Fig. 8b). However, overexpression of either LG5 or LG3/4/5 was effective to block cadmium-induced BTB disruption, maintaining the BTB integrity (Fig. 8a). Furthermore, overexpression of LG3/4/5 was capable of rescuing cadmium-mediated BTB disruption as noted in Fig. 8b. Subsequent studies have shown that LG5 or LG3/4/5 exerted their effects to block or rescue BTB function by maintaining or correcting distribution of TJ and basal ES proteins at the BTB (Fig. S1A, B), and also planar cell protein protein Dishevelled 3 (Dvl3, which was recently shown to modulate testis F-actin and MT organization^46^) (Fig. S2A, B). Collectively, these findings thus confirm unequivocally that LG3/4/5 (also LG5) is a potent biologically active peptide, capable of promoting spermatogenesis through its protective effects on BTB integrity.

## Discussion

Studies in vitro have shown that LG3/4/5 residing at the C-terminal region of the laminin-α2 chain is likely capable of promoting spermatogenesis and BTB function based on two recent reports^5,8^. This conclusion was reached based on observations that a knockdown of laminin-α2 impeded the Sertoli cell TJ-barrier function by perturbing the cytoskeletal organization of actin- and MT-based cytoskeletons across the Sertoli cell cytosol^5^. This finding is in agreement with studies performed in other study models. For instance, it was shown that a 16-amino acid peptide derived from human laminin-α5 chain, but not the corresponding peptide from laminin-α3 chain, was able to up-regulate the expression and activity of MMP-9 in mouse macrophages^47^. This and other findings thus support the notion that fragments of extracellular matrix are mediators of inflammation^48^. Also, a peptide, designated C16-peptide, derived from laminin-γ1 chain is a potent angiogenic peptide in vivo by exerting its effects through integrins^49^. Herein, we showed that this LG3/4/5 fragment promoted BTB function and spermatogenesis in the testis in vivo by utilizing the cadmium-mediated testis injury model. Interestingly, by deletion analysis, it was shown that the fragment designated LG5-peptide in the LG3/4/5 fragment contained most biological activity, but the whole LG3/4/5 fragment remains to be more effective in protecting cadmium-mediated Sertoli cell injury based on studies in vitro and also in vivo. Taking collectively, these findings suggest that the biological activity of the LG5-peptide in LG3/4/5 requires the participation of another stretch(es) of amino acid residues to exert its full biological activity. Nonetheless, these differences in results based the use of different cDNA constructs might be due to differences in transfection efficiency. In order to address this possibility, we had estimated the transfection efficiency in studies in vivo with testes, which was shown to be ~70% when PolyPlus in vivo-jetPEI® served as the transfection reagent. Studies in vitro using different cDNA constructs wherein plasmid DNA was labeled with Cys (red fluorescence) and using Lipojet In Vitro Transfection Reagent for transfection, the transfection efficiency was estimated to be at least 90%. Also, based on findings of in vitro studies when the expression levels of different cDNA constructs were assessed by RT-PCR and qPCR, their expression levels were relatively similar. Thus, it is unlikely that differences obtained by using different constructs were the result of differences in transfection efficiency.

Most importantly, our findings have shown that LG3/4/5 not only blocks cadmium-induced testis injury, but it also rescues cadmium-mediated testis injury. While the mechanism remains to be better understood, but it is likely that LG3/4/5 promotes the “resealing” of the disrupted BTB, which in turn supports re-initiation of spermatogenesis so that germ cells can repopulate the entire seminiferous epithelium. Indeed, this concept is supported by two lines of evidence based on earlier studies. First, studies have shown treatment of neonatal rats with diethylstilbestrol (DES, a synthetic estrogen)^50^ or adjudin (a non-hormonal male contraceptive that exerts its effects to perturb elongating/elongated spermatid adhesion)^51^ that delays BTB assembly also delays the onset of meiosis. For instance, spermatocytes in neonatal rats treated with DES without a functional BTB undergo apoptosis, failing to enter meiosis until the BTB is “resealed”^50^. Also, the establishment of a functional BTB by 15-17 dpp (days postpartum in rodents) is closely related to the onset of meiosis^52–54^. Second, rats treated with an acute dose of adjudin^55^, cadmium^45,56^ and glycerol^57^ that induced irreversible BTB damage in rodents also led to sterility^26,56,58–62^, illustrating the significance of a functional (and intact) BTB to support spermatogenesis. Furthermore, it was noted that cadmium-induced testis injury is mediated by disruptive changes on the cytoskeletal organization of F-actin and MTs, consistent with earlier reports that toxicants (e.g., 2,5-hexanedione, phthalates) perturb testis function through changes in cytoskeletal organization^63–66^. Interestingly, LG3/4/5 (also LG5) was found to exert its promoting effects via re-distribution of F-actin and MT regulatory proteins across the seminiferous epithelium, or changes in spatial expression of the corresponding cytoskeletal regulatory proteins. As such, the re-distributed actin and MT regulatory proteins are capable of re-organizing cytoskeletons across the epithelium to support spermatogenesis. Even though epithelial damage is extensive following treatment of rats with toxicants, such as CdCl_2_, overexpression of LG3/4/5 was able to re-build the seminiferous epithelium by coordinating proper organization of the F-actin and MT cytoskeletal networks to support spermatogenesis. On the other hand, it is noted that the protective effects provided by overexpression of LG3/4/5 was reduced somewhat when CdCl_2_ treatment was administered prior to LG3/4/5 overexpression (based on findings shown in Figs. 4, 5, 7, and S2). These findings suggest that some interacting functional proteins are eliminated at the Sertoli cell junctions by prior CdCl_2_ treatment, such that the subsequent overexpression of LG3/4/5 may not be able to interact with the missing proteins to better promoting spermatogenesis. In order to address this and other possibilities, bioinformatics studies based on RNA-Seq for transcriptome profiling from these groups should offer some helpful clues, which should be carefully evaluated in future studies.

Nonetheless, these findings suggest that overexpression of LG3/4/5 in the testis should be carefully evaluated as a therapeutic option to treat idiopathic male infertility, in particular in patients who had been exposed to environmental toxicants, such as among industrial workers requiring routine handling of toxicants.

## Supplementary information

Supplemental Figure Legends

Figure S1

Figure S2
