# Supplementary material for: Endogenously produced LG3/4/5-peptide protects testes against toxicant-induced injury
Source: Cell Death Dis. 2020 Jun 8;11(6):436. doi: 10.1038/s41419-020-2608-8 (PMC7280515; doi:10.1038/s41419-020-2608-8)
Supplement: Supplementary file 1 — Supplemental Figure Legends [file 41419_2020_2608_MOESM1_ESM.doc]

**Supplementary Material**

**Figure S1. LG5 and LG3/4/5 protect the BTB against cadmium-induced injury through changes in the distribution of BTB-associated proteins.** A study was performed to examine the promoting effects of LG5 *vs.* LG3/4/5 in blocking the cadmium-induced BTB disruption **(A)** and the rescuing effects of LG3/4/5 in cadmium-mediated BTB disruption **(B)**. The regimen used for the experiment is in the top panel of the corresponding experiment with *n*=4 rats per group. Results of the normal testes (-ve control) were not shown which were similar to the pCI-neo/Ctrl (another –ve control) as noted in **(B)**, and both sets of experiments were processed in the same experimental session. Cadmium treatment was found to perturb the location of TJ (CAR, ZO-1) and basal ES (N-cadherin, ß-catenin) located at the BTB near the basement membrane (annotated by the dashed white line) (see yellow blankets) considerably vs. control testes (annotated by white blankets) in **(A)** and **(B)**. However, LG5 or LG3/4/5 was effective to block cadmium-induced disruptive localization of these BTB-associated proteins (see white blankets), making them similar to the control testes (pCI-neo/Ctrl) (see white blankets). Scale bar, 40 µm, applies to both **(A)** and **(B)** for corresponding images.

**Figure S2. LG5 and LG3/4/5 protect testes against cadmium-induced injury through changes in the distribution of PCP protein Dvl3 and F-actin.** A study was performed to examine the effects of LG5 vs. LG3/4/5 in blocking the cadmium-induced testis injury **(A)** and the rescuing effects of LG3/4/5 in cadmium-mediated testis injury **(B)** through changes in PCP protein Dvl3 which recently showed to exert its effects through cytoskeletal organization [^6^](#_ENREF_6). The regimen used for the experiment is shown on the top panel of the corresponding experiment with *n*=4 rats per group. Results of the normal testes (-ve control) were not shown which were similar to the pCI-neo/Ctrl (another –ve control) as noted in **(B)**, and both sets of experiments were processed in the same experimental session. Dvl3 appeared as stalk-like structures that stretched across the seminiferous epithelium but also at the BTB (see red arrowheads) near the basement membrane (annotated by dashed white line) in the seminiferous epithelium, consistent with an earlier report [^6^](#_ENREF_6). However, in all CdCl_2_ treatment groups, unless LG5 or LG3/4/5 was overexpressed to block or rescue toxicant-induced testis injury, Dvl3 distribution was grossly perturbed. Scale bar, 80 µm, which applies to other micrographs in **(A)** or **(B)**.

**References**

**1. Talts, J.F., Mann K., Yamada Y., Timpl R. Structural analysis and proteolytic processing of recombinant G domain of mouse laminin α2 chain. *FEBS Lett* 426, 71-76 (1998).**

**2. Gao, Y., Lui W.Y., Lee W.M., Cheng C.Y. Polarity protein Crumbs homolog-3 (CRB3) regulates ectoplasmic specialization dynamics through its action on F-actin organization in Sertoli cells. *Sci Rep* 6, 28589 (2016).**

**3. O'Donnell, L., O'Bryan M.K. Microtubules and spermatogenesis. *Semin Cell Dev Biol* 30, 45-54 (2014).**

**4. Su, L., Mruk D.D., Lie P.P.Y., Silvestrini B., Cheng C.Y. A peptide derived from laminin-γ3 reversibly impairs spermatogenesis in rats. *Nat Communs* 3, 1185 (doi:1110.1038/ncomms2171) (2012).**

**5. Cheng, C.Y., Mruk D.D. The blood-testis barrier and its implication in male contraception. *Pharmacol Rev* 64, 16-64 (2012).**

**6. Li, L., Mao B., Yan M., Wu S., Ge R., Lian Q.*, et al.* Planar cell polarity protein Dishevelled 3 (Dvl3) regulates ectoplasmic specialization (ES) dynamics in the testis through changes in cytoskeletal organization. *Cell Death & Disease* 10:194 (DOI:10.1038/s41419-019-1394-7), (2019).**
